# Bioprocessed Black Rice Bran and Balloon Flower Root Cooperatively Regulate IgE, Epithelial Signaling, and Th1/Th2 Balance to Induce Therapeutic Response in a Mouse Model of Atopic Dermatitis

**DOI:** 10.3390/ijms27062691

**Published:** 2026-03-16

**Authors:** Kyung Hee Lee, Ki Sun Kwon, Woon Sang Hwang, Alan D. Friedman, Wha Young Lee, Jeanman Kim, Sang Jong Lee, Sung Phil Kim, Mendel Friedman

**Affiliations:** 1 STR Biotech Co., Ltd., Chuncheon 24232, Republic of Korea; 2 Department of Oncology, Johns Hopkins University School of Medicine, Baltimore, MD 21205, USA; 3 College of Pharmacy, Ajou University, Suwon 16499, Republic of Korea; 4 U.S. Department of Agriculture, Western Regional Research Center, Agricultural Research Service, Albany, CA 94710, USA

**Keywords:** atopic dermatitis, immune modulation, bioprocessed black rice bran, bioprocessed balloon flower root, Th1/Th2 balance, regulatory T cells

## Abstract

Atopic dermatitis (AD) is a chronic inflammatory skin disorder characterized by epidermal barrier dysfunction and dysregulated immune responses, particularly an imbalance between T helper type 1 (Th1) and type 2 (Th2) cytokines. Natural products with immunomodulatory activity have attracted increasing attention as potential strategies for regulating allergic inflammation. In this study, we investigated the immunomodulatory effects of bioprocessed black rice bran (BRB-F) and bioprocessed balloon flower root (BFR-F). In vitro assays using human B cells, mast cells, and keratinocytes were conducted to evaluate IgE production, mast cell degranulation, and epithelial inflammatory mediator release. The efficacy of the BRB-F:BFR-F mixture was further evaluated in BALB/c mice with 2,4-dinitrochlorobenzene (DNCB)/*Dermatophagoides farinae* extract (DFE)-induced AD-like dermatitis. BRB-F and BFR-F suppressed IgE production, attenuated mast cell degranulation and thymic stromal lymphopoietin (TSLP) release, and reduced keratinocyte-derived inflammatory mediators (thymus and activation-regulated chemokine (TARC), macrophage-derived chemokine (MDC), and IL-6). In mice, dietary supplementation with the BRB-F:BFR-F mixture (10–80 mg/kg/day) dose-dependently improved clinical skin lesions and histopathological changes, with serum IgE reduced by up to 87.1% at the highest dose. The treatment significantly suppressed Th2 cytokine mRNA expression in ear tissue (IL-4, IL-5, and IL-13) by 37.2%, 32.7%, and 34.0%, respectively, compared with the positive control. In contrast, splenic Th1 cytokine mRNA expression (IL-2, IL-12, and IFN-γ) was partially restored by 37.1%, 22.5%, and 18.7%, respectively. These findings indicate that BRB-F and BFR-F modulate multiple immune pathways and help restore Th1/Th2 immune balance, suggesting their potential as functional materials for regulating immune dysregulation associated with AD.

## 1. Introduction

Atopic dermatitis (AD) is a chronic inflammatory skin disease characterized by recurrent eczematous lesions, intense pruritus, and substantial impairment of quality of life [1]. The pathogenesis of AD is multifactorial and involves complex interactions among genetic susceptibility, epidermal barrier dysfunction, gut microbial dysbiosis, and dysregulated immune responses [2,3]. Immunologically, AD is marked by dominant type 2 immune activation, including elevated Th2 cytokines, enhanced IgE production, mast cell degranulation, and aberrant keratinocyte-derived cytokine and chemokine signaling [1,4]. These immune abnormalities extend beyond the skin and are associated with systemic immune alterations. They are also linked to multiple comorbid allergic and inflammatory conditions [5]. AD is also one of the most common chronic inflammatory skin diseases worldwide, affecting approximately 15–20% of children and 1–3% of adults [3]. The disease most frequently develops during early childhood, although symptoms may persist into adolescence and adulthood in a substantial proportion of patients.

Despite major advances in pharmacological therapies, including biologics targeting IL-4 and IL-13 signaling pathways, long-term management of AD remains challenging [6]. Many patients experience incomplete disease control, treatment-related adverse effects, or relapse after treatment discontinuation, underscoring the need for complementary approaches that support immune homeostasis rather than single-pathway suppression [7].

Functional dietary materials have therefore gained increasing attention as adjunctive strategies for chronic inflammatory disorders such as AD [8]. Conventional pharmacological treatments may be limited by safety concerns during prolonged use, particularly in pediatric and elderly populations [6,9]. In contrast, food-derived bioactive materials may exert broad immunomodulatory effects while maintaining favorable safety profiles during long-term administration [10].

Importantly, dietary components are capable of modulating multiple immune and epithelial targets relevant to AD pathophysiology, including B-cell-mediated IgE production, mast cell activation, keratinocyte-derived cytokine release, and T-cell polarization [11,12]. Such multi-target immunomodulation is increasingly recognized as a desirable feature for restoring immune balance in complex inflammatory diseases [8,13].

Black rice bran is a byproduct of rice milling that contains diverse bioactive compounds, including polysaccharides, phenolic acids, and anthocyanins, which have been reported to exhibit anti-inflammatory and antioxidant activities in experimental models [14]. Early work demonstrated that non-bioprocessed black rice bran can protect against chemically induced inflammatory skin responses in mice, indicating that the raw material itself possesses biologically relevant activity [15].

Building on this baseline activity, subsequent studies showed that black rice bran bioprocessed with shiitake (*Lentinus edodes*) mycelia yields preparations with reproducible biological effects across multiple experimental systems [16]. Bioprocessed black rice bran has been reported to modulate systemic inflammatory challenges, including endotoxemia [16], allergic asthma [17], metabolic inflammation [18], and immune checkpoint-associated antitumor responses in vivo [19]. Collectively, these findings indicate that fermentation-driven modification substantially alters the functional properties of black rice bran.

Mechanistically, the enhanced activity of bioprocessed black rice bran has been attributed to enzymatic degradation of complex polysaccharide structures, release of bound phenolic compounds, and generation of lower-molecular-weight bioactive fractions with improved biological accessibility [20,21]. Thus, bioprocessing does not negate the intrinsic activity of black rice bran but rather amplifies and stabilizes its functional performance across experimental models.

Balloon flower root (*Platycodon grandiflorum*) is an edible plant material containing multiple bioactive constituents, most notably triterpenoid saponins such as platycodin D and platycodin D2, as well as polysaccharides. Experimental studies using non-bioprocessed extracts have demonstrated that *P. grandiflorum*-derived components exert anti-inflammatory and immunomodulatory effects in a variety of cell-based and animal models [22,23,24]. These activities have been linked to modulation of inflammatory cytokine production, oxidative stress responses, endothelial function, and intracellular signaling pathways.

An important methodological consideration in the development of plant-derived functional materials is whether and how bioprocessing enhances biological performance beyond that observed for non-processed counterparts. Bioprocessing approaches, including microbial fermentation and enzymatic treatment, have been shown to modify plant matrices by reducing molecular complexity, increasing solubility, and generating bioactive derivatives with improved functional accessibility [20,25]. Consistent with this concept, recent studies have reported that hydrolyzed and fermented *Platycodon grandiflorum* extracts exhibit enhanced immunomodulatory activity through modulation of intracellular signaling pathways, including the MAPK and NF-κB signaling pathways [26]. For polysaccharide- and saponin-rich materials, such processing may also enhance biological relevance by facilitating interaction with immune cells, modulating gut microbial metabolism, and improving batch-to-batch reproducibility [21,25].

Atopic dermatitis involves coordinated dysregulation across multiple immune and epithelial compartments, including B-cell-mediated IgE production, mast cell activation, keratinocyte-derived cytokine release, and imbalance between Th2- and Th1-associated immune responses [1,4]. Given this multifaceted pathology, functional materials that influence distinct but complementary immunological pathways may offer advantages over single-component interventions. Evaluating bioprocessed black rice bran and balloon flower root in combination therefore provides an opportunity to assess whether coordinated modulation of multiple immune axes can be achieved within a single functional formulation [8,13].

Accordingly, we hypothesized that coordinated modulation of B-cell IgE production, mast cell activation, keratinocyte-derived inflammatory signaling, and Th1/Th2 imbalance may contribute to the restoration of immune homeostasis in AD. Rather than targeting a single cytokine pathway, such multi-axis immune engagement could provide a broader regulatory effect in complex inflammatory conditions.

The present study was therefore designed to systematically evaluate the mechanistic effects of bioprocessed black rice bran and balloon flower root, administered individually and as a defined binary formulation, in established cellular systems and a murine model of atopic dermatitis, with particular emphasis on IgE regulation, mast cell and epithelial activation, Th1/Th2 polarization, and Treg-associated immune markers.

## 2. Results

### 2.1. Inhibitory Effects of BRB-F and BFR-F on IgE Production in Human U266.B1 Cells

To evaluate the ability of bioprocessed black rice bran (BRB-F) and bioprocessed balloon flower root (BFR-F) extracts to suppress IgE production, assays were conducted using the human B-cell line U266.B1. Both individual extracts and their combinations were examined. Stimulation of U266.B1 cells with lipopolysaccharide (LPS) and interleukin-4 (IL-4) increased IgE secretion by more than 50-fold compared with unstimulated controls. Treatment with the raw extracts, BRB and BFR, led to modest reductions in IgE production, with BRB decreasing IgE levels by 18.1% and BFR by 10.7% relative to the positive control. In contrast, the bioprocessed extracts BRB-F and BFR-F exhibited markedly stronger inhibitory activity, each suppressing IgE production by approximately 60%. Combinations of BRB-F and BFR-F at different ratios (3:1, 1:1, and 1:3) resulted in inhibitory levels comparable to those observed with each extract alone, with no significant differences among mixing ratios (Table 1).

These findings indicate that bioprocessing substantially enhances the capacity of black rice bran and balloon flower root to suppress B-cell-mediated IgE production. The comparable effects observed across different mixing ratios suggest that IgE regulation is primarily driven by the intrinsic activity of each bioprocessed component rather than by ratio-dependent synergistic interaction. This early modulation of IgE production represents a potential upstream mechanism for attenuating allergic effector responses in atopic dermatitis.

### 2.2. Degranulation Inhibitory Effects of BRB-F and BFR-F in RBL-2H3 Mast Cells

The inhibitory activity of bioprocessed black rice bran (BRB-F) and bioprocessed balloon flower root (BFR-F) on mast cell degranulation was evaluated using the RBL-2H3 rat basophilic leukemia cell line. Both individual and combined treatments were tested to determine their effects on β-hexosaminidase release, a key marker of mast cell degranulation. Stimulation with A23187 induced a strong degranulation response. Treatment with the raw materials, BRB and BFR, reduced β-hexosaminidase release by 52.7% and 31.5%, respectively, compared with the positive control. BRB-F and BFR-F also inhibited degranulation, decreasing β-hexosaminidase release by 58.4% and 33.9%, respectively. When BRB-F and BFR-F were combined at different mixing ratios, the degree of inhibition was comparable to that produced by BRB-F alone, and no significant differences were detected among the tested combinations (Table 2).

These results demonstrate that bioprocessed BRB-F retains strong inhibitory activity against mast cell degranulation, a central effector event in allergic inflammation. The lack of significant ratio-dependent differences among combined treatments suggests that mast cell stabilization is primarily attributable to the dominant activity of BRB-F within the formulation. Together with the suppression of IgE production observed in B cells, these findings indicate that the formulation modulates both upstream antibody production and downstream effector cell activation within the allergic response cascade.

### 2.3. Evaluation of BRB-F and BFR-F Inhibitory Effects on TSLP Production in HMC-1.2 Cells

The inhibitory effects of bioprocessed black rice bran (BRB-F) and bioprocessed balloon flower root (BFR-F) on thymic stromal lymphopoietin (TSLP) production were examined using the human mast cell line HMC-1.2. Both individual and combined treatments were assessed to determine their relative efficacy. Stimulation with PMA and A23187 elevated TSLP production by 2.3-fold compared with baseline. Treatment with the raw materials BRB and BFR reduced TSLP levels by 34.5% and 18.9%, respectively. In contrast, treatment with the bioprocessed forms resulted in greater suppression, with BRB-F reducing TSLP production by 51.9% and BFR-F by 31.3% (Table 3). Combination treatments of BRB-F and BFR-F showed that increasing the proportion of BRB-F produced slightly stronger TSLP inhibition, although the degree of suppression was comparable among the tested mixing ratios.

TSLP is a key epithelial-derived alarmin that bridges innate and adaptive type 2 immune responses by promoting Th2 polarization and IgE-associated inflammation [1]. The stronger inhibitory activity of BRB-F compared with the raw material further supports the role of bioprocessing in enhancing functional accessibility of immunoregulatory components. Collectively, the suppression of TSLP production, together with the inhibition of IgE secretion and mast cell degranulation, indicates coordinated modulation of early allergic signaling pathways relevant to AD pathophysiology.

### 2.4. Inhibitory Effects of BRB-F and BFR-F on Cytokine and Chemokine Expression in HaCaT Keratinocyte Cells

The inhibitory effects of bioprocessed black rice bran (BRB-F) and bioprocessed balloon flower root (BFR-F) on cytokine and chemokine production were evaluated in human HaCaT keratinocyte cells. Both individual and combined treatments were examined to determine their relative efficacy. Stimulation with TNF-α and IFN-γ elevated the expression of TARC, MDC, and IL-6 by 29-fold, 89-fold, and 17-fold, respectively. Treatment with the raw materials BRB and BFR significantly suppressed these induced levels, with BRB showing greater inhibitory activity than BFR by 1.7-fold for TARC, 2.2-fold for MDC, and 1.5-fold for IL-6. Among the bioprocessed materials, BFR-F exhibited higher inhibitory potency than BRB-F, producing 1.5-fold, 1.2-fold, and 0.8-fold stronger suppression of TARC, MDC, and IL-6, respectively. Combination treatments of BRB-F and BFR-F resulted in inhibition levels comparable to those of the bioprocessed extracts alone (Table 4).

Keratinocyte-derived chemokines such as TARC and MDC are critical mediators of Th2 cell recruitment and amplification of cutaneous type 2 inflammation [1]. The marked suppression of these mediators by the bioprocessed extracts indicates effective modulation of epithelial inflammatory signaling, a central component of AD pathophysiology. When considered together with the inhibition of IgE production, mast cell degranulation, and TSLP release, these findings demonstrate coordinated regulation of multiple early immune and epithelial pathways involved in allergic inflammation.

Based on these coordinated effects observed in cellular systems, we next examined whether oral administration of the BRB-F:BFR-F formulation modulates immune responses and disease severity in an in vivo model of AD.

### 2.5. Anti-Atopic Effects of BRB-F and BFR-F in a Mouse Model of Atopic Dermatitis

**Figure 1:**
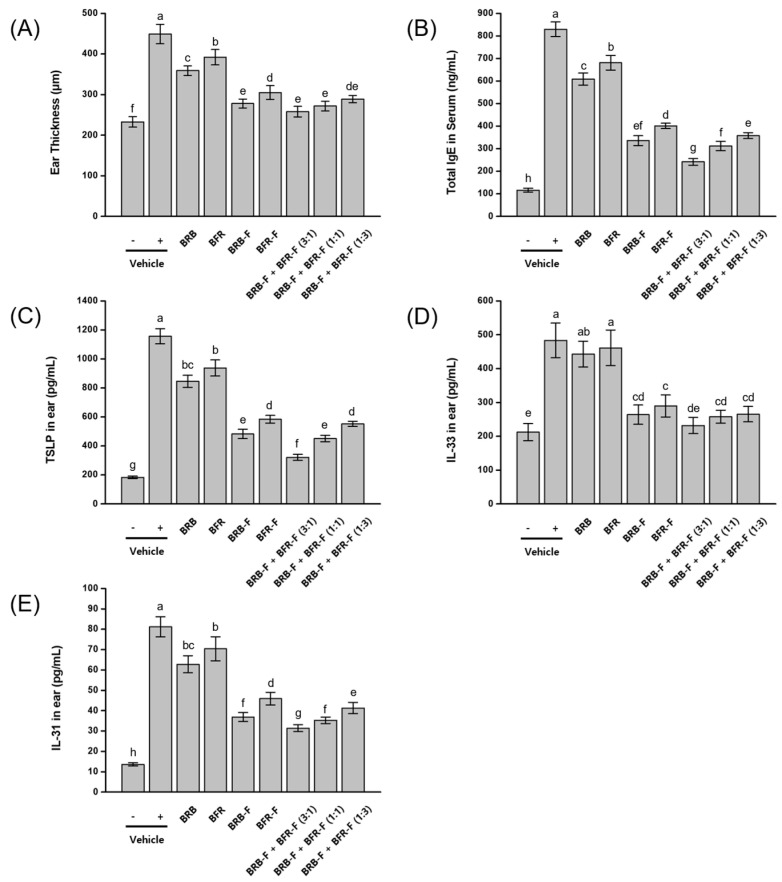
Effect of the combined ratio of BRB-F and BFR-F on DNCB- and DFE-induced atopic dermatitis. Atopic dermatitis was induced by DNCB (1%, 20 μL) and DFE (*Dermatophagoides farinae* extract) ointment (100 mg/mL) applied weekly to the right ear for 5 weeks; one week after the initial challenge, test samples were administered orally for 4 weeks. (**A**) Ear thickness was assessed using a micrometer. (**B**) Serum level of IgE was measured using ELISA. (**C**) TSLP, (**D**) IL-33, and (**E**) IL-31 in ear skin were measured by ELISA. Groups: vehicle (-), negative control not stimulated with DNCB and DFE; vehicle (+), DNCB- and DFE-stimulated positive control; BRB, black rice bran water extracts (40 mg/kg body weight); BFR, Balloon flower root water extract (40 mg/kg body weight); BRB-F, bioprocessed black rice bran extract (40 mg/kg body weight); BFR-F, bioprocessed balloon flower root extract (40 mg/kg body weight); BRB-F:BFR-F (3:1), bioprocessed black rice bran extract (30 mg/kg body weight) and bioprocessed balloon flower root extract (10 mg/kg body weight); BRB-F:BFR-F (1:1), bioprocessed black rice bran extract (20 mg/kg body weight) and bioprocessed balloon flower root extract (20 mg/kg body weight); BRB-F:BFR-F (1:3), bioprocessed black rice bran extract (10 mg/kg body weight) and bioprocessed balloon flower root extract (30 mg/kg body weight). Results are presented as mean ± SD (*n* = 10). Bars sharing a common letter are not significantly different between groups at *p* < 0.05.

The therapeutic effects of bioprocessed black rice bran (BRB-F) and bioprocessed balloon flower root (BFR-F), administered individually or in combination, were evaluated in a mouse model of atopic dermatitis (AD) induced by repeated exposure to 2,4-dinitrochlorobenzene (DNCB) and DFE (*Dermatophagoides farinae* extract). Disease severity was assessed by measuring ear thickness, serum IgE levels, and the expression of thymic stromal lymphopoietin (TSLP), interleukin-33 (IL-33), and interleukin-31 (IL-31) in ear tissue. In AD-induced mice, ear thickness, serum IgE, TSLP, IL-33, and IL-31 levels were markedly elevated compared with normal controls. Treatment with the bioprocessed materials resulted in significantly greater reductions in these markers than their corresponding raw forms. Among the individual treatments, BRB and BRB-F consistently produced stronger inhibitory effects than BFR and BFR-F. Combination treatments of BRB-F and BFR-F further reduced these biomarkers, with inhibitory efficacy increasing as the proportion of BRB-F increased in the mixture. The greatest therapeutic effect was observed for the 3:1 BRB-F:BFR-F ratio (30 mg/kg BRB-F and 10 mg/kg BFR-F), which reduced ear thickness to 84.5% of the control level, serum IgE to 82.4%, TSLP expression to 85.9%, IL-33 to 92.9%, and IL-31 to 73.7% (Figure 1).

These in vivo findings extend the cellular observations by demonstrating that oral administration of the BRB-F:BFR-F formulation attenuates both local cutaneous inflammation and systemic IgE-associated responses. The suppression of epithelial alarmins (TSLP, IL-33, IL-31) in ear tissue is consistent with reduced Th2 amplification within the skin microenvironment, while the reduction in serum IgE mirrors the inhibition of B-cell IgE production observed in vitro. The enhanced efficacy observed with increasing proportions of BRB-F suggests that this component plays a dominant role in regulating early inflammatory and type 2 immune pathways in the AD model.

### 2.6. Modulation of Th2 and Th1 Cytokine Expression by BRB-F and BFR-F in Atopic Dermatitis

Th2 cells are known to predominate in lesioned tissues following the induction of atopic dermatitis (AD) [27]. To evaluate immune modulation in this model, the mRNA expression levels of Th2 cytokines (IL-4, IL-5, and IL-13) were measured in ear tissue. AD induction resulted in strong upregulation of these cytokines, exceeding a 5-fold increase compared with normal, untreated mice. Administration of the bioprocessed extracts, either alone or in combination, produced greater suppression of Th2 cytokine expression than the corresponding raw materials. The most pronounced inhibitory effect was observed with the 3:1 BRB-F:BFR-F combination, which reduced IL-4 mRNA to 48.8%, IL-5 to 49.1%, and IL-13 to 49.7% of the AD-induced control (Table 5). To further examine T-cell-mediated immune responses, expression levels of the Th1 cytokines IL-2, IL-12, and IFN-γ were measured in spleen tissue. AD induction markedly decreased Th1 cytokine expression. Treatment with the bioprocessed materials, particularly the 3:1 BRB-F:BFR-F combination, substantially restored Th1 cytokine levels, with recovery most notable for IL-2 (68.4% of normal), followed by IL-12 (48.3% of normal) and IFN-γ (76% of normal).

These results demonstrate that oral administration of the BRB-F:BFR-F formulation not only suppresses cutaneous Th2-skewed inflammation but also promotes partial restoration of Th1-associated cytokine expression in systemic lymphoid tissue. Such bidirectional modulation suggests rebalancing of the Th1/Th2 axis rather than simple inhibition of type 2 responses. When considered together with the attenuation of IgE production, mast cell activation, and epithelial alarmin expression, these findings support coordinated regulation of both local and systemic immune pathways in the AD model.

### 2.7. Effects of BRB-F and BFR-F on IL-10 Cytokine Expression in Atopic Dermatitis

**Figure 2:**
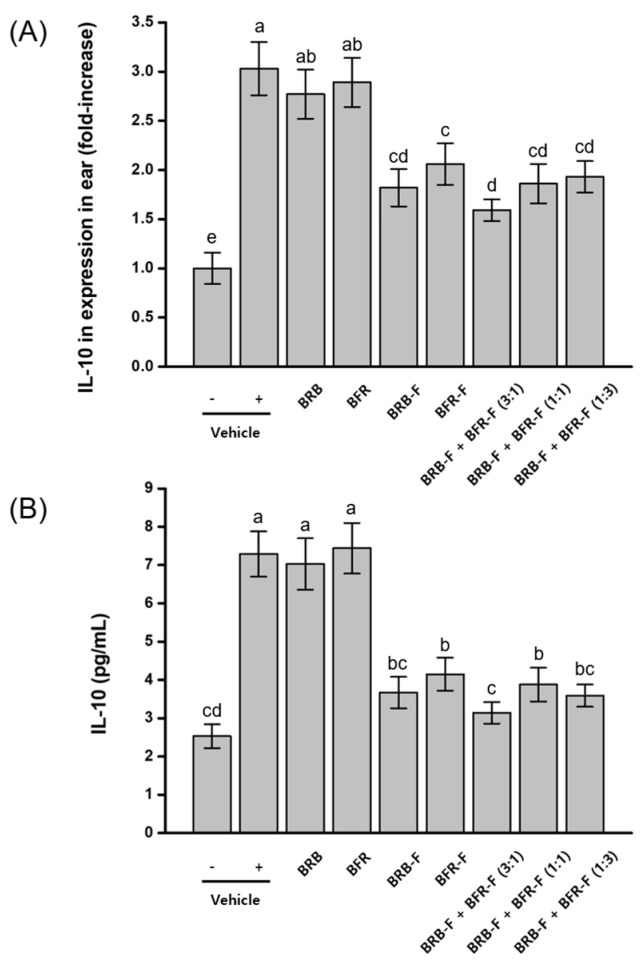
Effects of the combined ratio of BRB-F and BFR-F on the IL-10 expression. The IL-10 in ears was measured by RT-PCR intensity (**A**) and ELISA (**B**), shown for atopic dermatitis. Results are presented as mean ± SD (*n* = 10). Bars sharing a common letter are not significantly different between groups at *p* < 0.05.

Previous studies have reported that IL-10 can be induced during Th2-dominant inflammatory responses associated with atopic dermatitis [28]. To evaluate this mechanism, IL-10 mRNA and protein expression levels were measured in ear tissue. AD induction resulted in an approximately threefold increase in both IL-10 mRNA and protein expression compared with normal, untreated mice. Administration of the bioprocessed materials produced greater inhibition of IL-10 expression than their raw counterparts. Among the treatment groups, the combination of BRB-F and BFR-F at a 3:1 ratio yielded the strongest effect, reducing IL-10 mRNA expression by 87.2% and protein expression by 70.9% relative to the AD-induced group (Figure 2).

Although IL-10 is generally recognized as a regulatory cytokine, elevated IL-10 levels have also been reported in Th2-dominant inflammatory environments, where its role may reflect compensatory or dysregulated immune responses [28]. The reduction in IL-10 expression observed in the present study likely reflects normalization of exaggerated cytokine signaling rather than suppression of protective regulatory pathways. When interpreted alongside the restoration of Th1-associated cytokines and the modulation of epithelial and IgE-related responses, these findings suggest reorganization of the inflammatory milieu toward a more balanced immune state rather than unidirectional immune suppression.

### 2.8. Effects of BRB-F and BFR-F on Galectin-9 Expression and Treg Activation in Atopic Dermatitis

**Figure 3:**
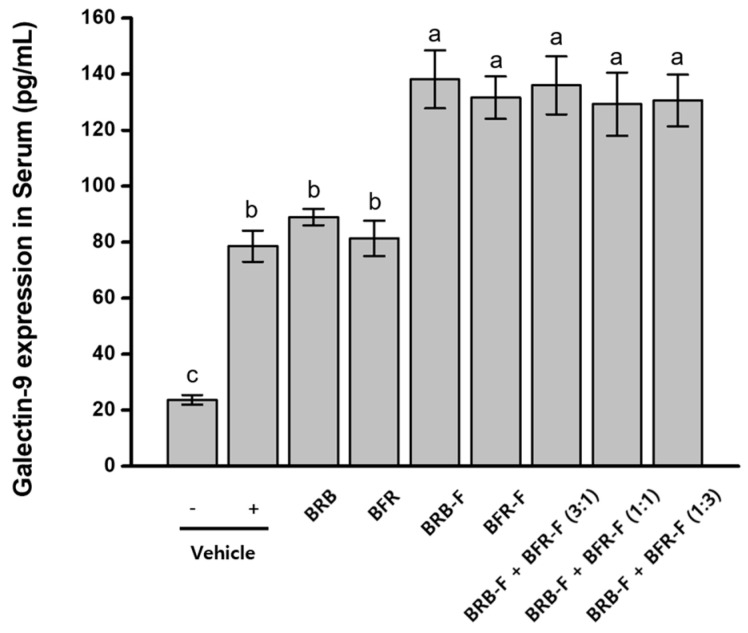
Effects of the combined ratio of BRB-F and BFR-F on the galectin-9 expression. Galectin-9 in serum was measured by ELISA. Results are presented as mean ± SD (*n* = 10). Bars sharing a common letter are not significantly different between groups at *p* < 0.05.

Galectin-9 plays a key role in immune regulation and has been reported to promote Treg-associated responses and modulate Th1/Th2 immune balance [29]. To evaluate whether BRB-F and BFR-F modulate this pathway, serum galectin-9 levels were measured in mice treated with the extracts individually or in combination. Mice with induced atopic dermatitis exhibited a substantial increase in serum galectin-9 levels (78.59 ± 5.58 pg/mL), approximately 3.3-fold higher than those in normal controls (23.67 ± 1.73 pg/mL). Administration of BRB-F and BFR-F further elevated serum galectin-9 levels, and no significant differences were observed between single-agent and combination treatments (Figure 3).

Galectin-9 is recognized as an immunoregulatory molecule capable of promoting Treg-associated responses and modulating Th1/Th2 balance [29]. The further elevation of galectin-9 following administration of the bioprocessed extracts may reflect reinforcement of regulatory signaling pathways within the inflammatory environment. Notably, this increase occurred alongside suppression of Th2 cytokines and partial restoration of Th1-associated responses, suggesting that enhanced galectin-9 expression contributes to coordinated immune rebalancing rather than generalized immune activation. The comparable responses observed between single-agent and combination treatments further indicate that regulatory pathway engagement represents a shared property of the bioprocessed materials.

### 2.9. Evaluation of Dose-Dependent Effects of BRB-F and BFR-F Mixture on Atopic Dermatitis

**Figure 4:**
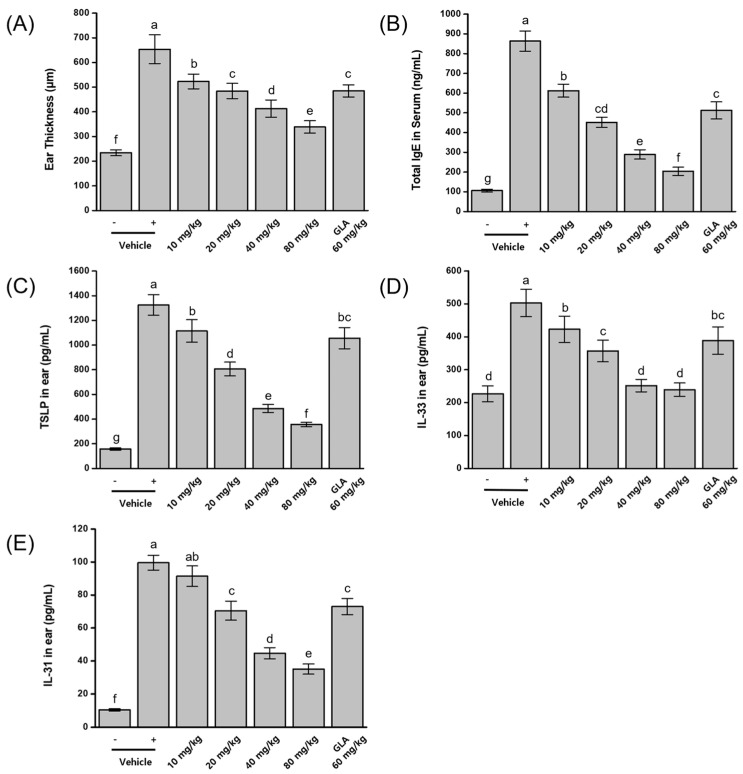
Dose-dependent effects of BRB-F and BFR-F (3:1) on DNCB- and DFE-induced atopic dermatitis. (**A**) Ear thickness was assessed using a micrometer. (**B**) Serum level of IgE was measured using ELISA. (**C**) TSLP, (**D**) IL-33 and (**E**) IL-31 in ear skin were measured by ELISA. Groups: vehicle (-), negative control not stimulated with DNCB and DFE; vehicle (+), DNCB- and DFE-stimulated positive control; 10 mg/kg, 3 bioprocessed black rice bran extract + 1 bioprocessed balloon flower root extract (10 mg/kg body weight); 20 mg/kg, 3 bioprocessed black rice bran extract + 1 bioprocessed balloon flower root extract (20 mg/kg body weight); 40 mg/kg, 3 bioprocessed black rice bran extract + 1 bioprocessed balloon flower root extract (40 mg/kg body weight); 80 mg/kg, 3 bioprocessed black rice bran extract + 1 bioprocessed balloon flower root extract (80 mg/kg body weight); GLA 60 mg/kg, γ-linolenic acid (GLA, 60 mg/kg body weight). Results are presented as mean ± SD (*n* = 10). Bars sharing a common letter are not significantly different between groups at *p* < 0.05.

To identify the optimal dosage for suppressing atopic dermatitis (AD), the bioprocessed black rice bran (BRB-F) and bioprocessed balloon flower root (BFR-F) mixture was evaluated at the previously determined optimal ratio of 3:1 (30 mg/kg BRB-F and 10 mg/kg BFR-F). Mice were administered daily oral doses of 10, 20, 40, and 80 mg/kg of the mixture following AD induction with DFE and DNCB. Disease severity was evaluated by measuring ear thickness, serum IgE levels, and the expression of TSLP, IL-33, and IL-31 in ear tissue. All biomarkers were significantly elevated in AD-induced mice compared with normal controls. Treatment with the BRB-F:BFR-F mixture produced dose-dependent suppression of AD symptoms. The highest dose (80 mg/kg) reduced ear thickness by 75%, serum IgE by 87.1%, TSLP by 82.9%, IL-33 by 95.4%, and IL-31 by 72.4% relative to the AD control (Figure 4). When compared with a reference group receiving 60 mg/kg γ-linolenic acid (GLA), the 20 mg/kg BRB-F:BFR-F treatment produced comparable or greater inhibition across multiple biomarkers.

The clear dose-dependent attenuation of both clinical and immunological markers further supports the functional robustness of the BRB-F:BFR-F formulation in modulating AD-associated inflammation. The parallel suppression of epithelial alarmins and systemic IgE across increasing doses suggests consistent regulation of early allergic signaling pathways. Notably, the comparable or superior efficacy observed relative to γ-linolenic acid indicates that the multi-target immune modulation achieved by this formulation may provide broader regulatory effects than single-pathway nutritional interventions.

### 2.10. Regulation of Th1/Th2 Immune Response by BRB-F and BFR-F Mixture

To evaluate the effects of the BRB-F and BFR-F mixture on Th1/Th2 immune regulation in atopic dermatitis (AD), mRNA expression levels of key cytokines were measured in mice. Th2 cytokines (IL-4, IL-5, and IL-13) were analyzed in lesional ear tissue, while Th1 cytokines (IL-2, IL-12, and IFN-γ) were quantified in spleen tissue using RT-PCR. AD induction resulted in a marked elevation of Th2 cytokine expression, with IL-4, IL-5, and IL-13 increasing more than fivefold compared with normal mice. Treatment with the BRB-F:BFR-F mixture produced dose-dependent suppression of Th2 cytokine expression, with the strongest effect observed at the highest dose (80 mg/kg), reducing IL-4, IL-5, and IL-13 by 45.3%, 39.2%, and 41.1%, respectively. AD induction also caused significant reductions in the expression of Th1-associated cytokines in the spleen. Administration of the BRB-F:BFR-F mixture resulted in dose-dependent restoration of Th1 cytokine levels, with the highest dose (80 mg/kg) increasing IL-2 to 60.5% of normal, IL-12 to 55.2%, and IFN-γ to 56% (Table 6). The 20 mg/kg dose of the BRB-F:BFR-F mixture produced levels of cytokine suppression and recovery comparable to those observed in the reference group treated with 60 mg/kg γ-linolenic acid (GLA).

The dose-dependent suppression of Th2 cytokines in lesional tissue, coupled with restoration of Th1-associated cytokine expression in the spleen, further supports the capacity of the BRB-F:BFR-F formulation to rebalance systemic immune responses in AD. Rather than exerting unidirectional immunosuppression, the formulation appears to normalize Th1/Th2 polarization in a graded manner across increasing doses. The comparable efficacy observed at 20 mg/kg relative to γ-linolenic acid highlights the potential of multi-target immune modulation to achieve regulatory effects at moderate dosing levels.

### 2.11. Inhibitory Effects of BRB-F and BFR-F Mixture on IL-10 Expression in Atopic Dermatitis-Induced Mice

**Figure 5:**
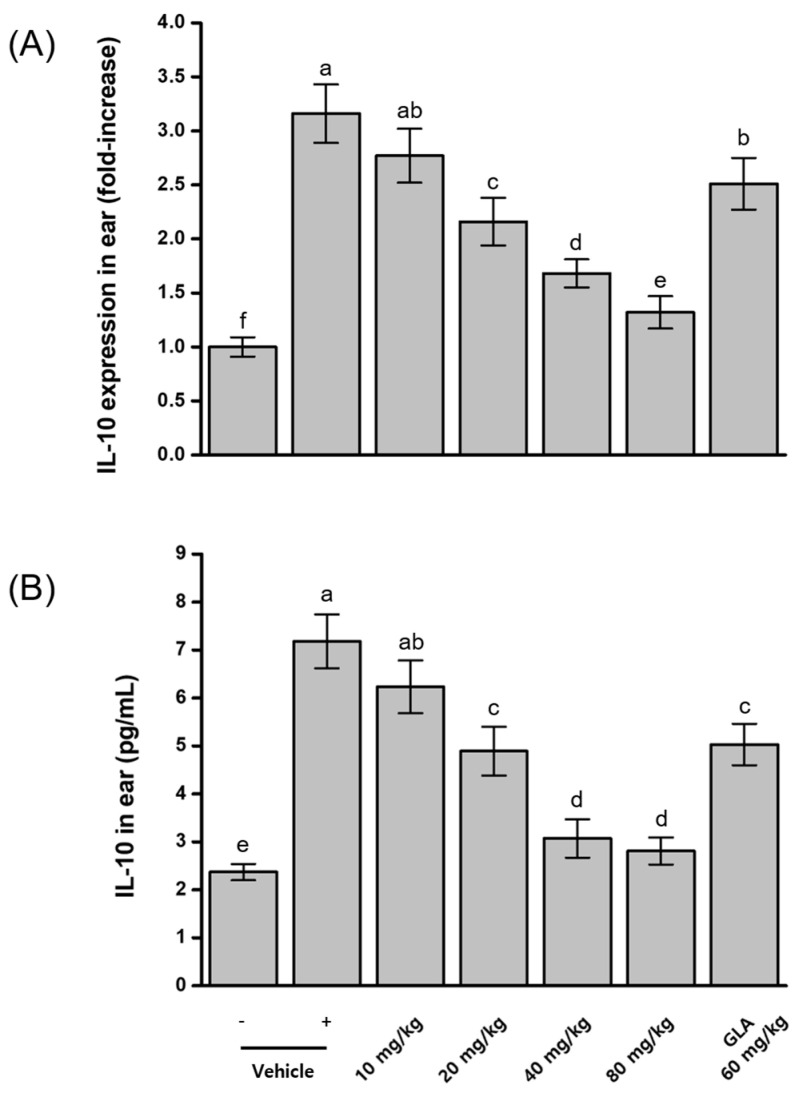
Dose-dependent effects of BRB-F and BFR-F (3:1) on the IL-10 expression. The IL-10 in ears was measured by RT-PCR intensity (**A**) and ELISA (**B**), shown for atopic dermatitis. Results are presented as mean ± SD (*n* = 10). Bars sharing a common letter are not significantly different between groups at *p* < 0.05.

Measurements of IL-10 mRNA and protein expression in ear tissue revealed significant overexpression of IL-10 in mice with induced atopic dermatitis. Administration of the BRB-F:BFR-F mixture produced dose-dependent suppression of IL-10 expression. The highest dietary dose (80 mg/kg) resulted in the greatest inhibition, reducing IL-10 mRNA by 90.9% and IL-10 protein by 85.2% relative to the AD-induced control (Figure 5). A comparative analysis with γ-linolenic acid (GLA) showed that the 20 mg/kg BRB-F:BFR-F treatment produced stronger IL-10 inhibitory activity than 60 mg/kg GLA.

The graded reduction in IL-10 expression across increasing doses is consistent with normalization of exaggerated cytokine signaling in the AD microenvironment. Although IL-10 is commonly regarded as an anti-inflammatory mediator, its elevated expression in Th2-dominant inflammatory settings may reflect dysregulated immune feedback. The strong dose-dependent modulation observed with the BRB-F:BFR-F mixture, together with restoration of Th1-associated cytokines and enhanced galectin-9 expression, suggests coordinated immune reorganization rather than indiscriminate suppression of regulatory pathways.

### 2.12. Dose-Dependent Effects of BRB-F and BFR-F Mixture on Galectin-9 Expression

**Figure 6:**
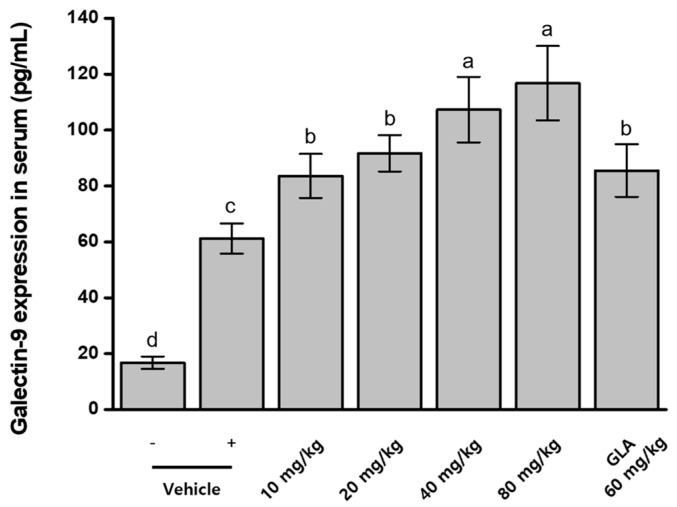
Dose-dependent effects of BRB-F and BFR-F (3:1) on the galectin-9 expression. Galectin-9 in serum was measured by ELISA. Results are presented as mean ± SD (*n* = 10). Bars sharing a common letter are not significantly different between groups at *p* < 0.05.

To assess the regulatory effects of the BRB-F:BFR-F mixture on galectin-9 expression, serum galectin-9 levels were measured in mice with induced atopic dermatitis (AD). ELISA analysis showed that AD induction resulted in a substantial elevation of serum galectin-9 (61.2 ± 5.4 pg/mL), approximately 3.6-fold higher than that of normal control mice (16.8 ± 2.2 pg/mL) (Figure 6). Administration of the BRB-F:BFR-F mixture produced a dose-dependent increase in galectin-9 expression, yielding 1.4-, 1.5-, 1.8-, and 1.9-fold increases in serum galectin-9 levels at incremental doses. When compared with γ-linolenic acid (GLA), the 10 mg/kg dose of the mixture exhibited comparable stimulatory activity to 60 mg/kg GLA, while higher doses surpassed the effect of GLA.

The progressive elevation of galectin-9 across increasing doses further supports activation of regulatory immune pathways by the BRB-F:BFR-F formulation. Although galectin-9 was elevated in AD-induced mice, the additional increase observed following treatment may reflect reinforcement of regulatory signaling within an inflammatory milieu. Importantly, this dose-dependent augmentation occurred alongside suppression of Th2 cytokines and partial restoration of Th1-associated responses, suggesting coordinated immune rebalancing rather than isolated pathway activation. The superior response at higher doses compared with γ-linolenic acid further highlights the broad immunomodulatory capacity of the formulation.

### 2.13. Histopathological Analysis of Ear Skin in Atopic Dermatitis Mice

**Figure 7:**
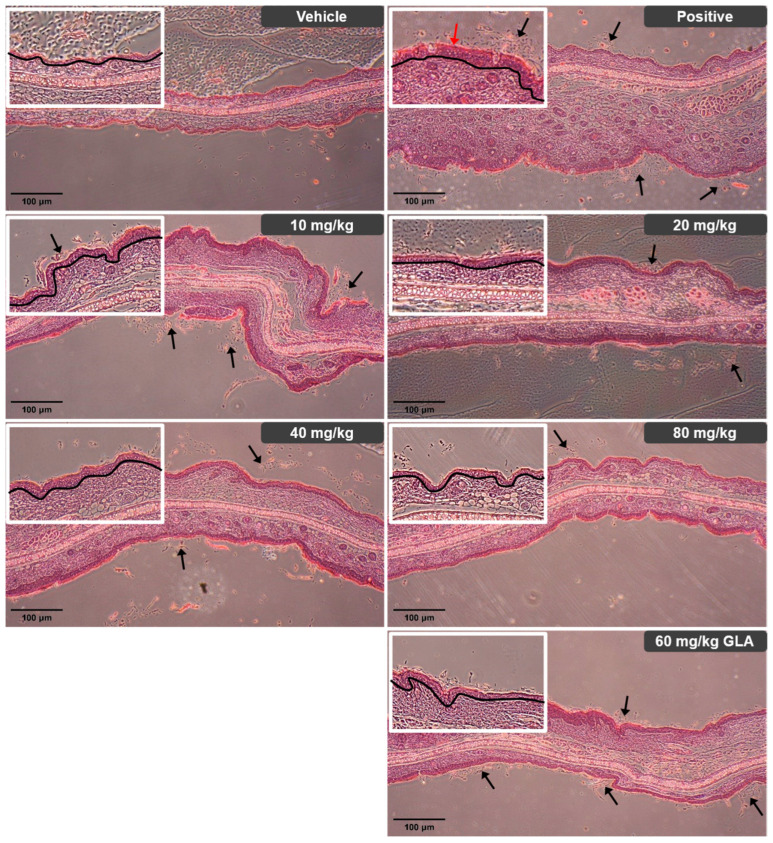
Histopathological analysis of ear skin in mice with atopic dermatitis using H&E staining. Ear tissues from AD BALB/c mice were fixed with 10% (*v*/*v*) paraformaldehyde. The fixed tissues were sectioned to 4 μm, followed by staining with hematoxylin and eosin (H&E) and light microscopy (magnification, ×40). Representative H&E-stained images of ear skin lesions from each group at week 5 are shown. The black line indicates ear thickness and epidermal thickness (hyperplasia). The red arrow indicates hyperkeratosis. The black arrows indicate exfoliating keratinocytes.

Histological examination of normal mice showed a thin epidermis composed of one to two cell layers, with minimal desquamation of the stratum corneum. In contrast, mice with induced atopic dermatitis (AD) displayed marked epidermal thickening characterized by multiple cell layers, increased numbers of blood vessels, sebaceous glands, hair follicles, and muscle bundles. The granular layer contained abundant keratohyalin granules, and the stratum corneum appeared uneven with extensive desquamation throughout the tissue [30,31]. Dietary administration of the BRB-F:BFR-F mixture at doses of 10, 20, 40, and 80 mg/kg produced dose-dependent improvements in these pathological alterations. Treatment resulted in progressive thinning of the epidermis relative to AD-induced mice, along with reduced granular layer thickness. The stratum corneum became more continuous and uniform, with minor areas of partial desquamation remaining. The most substantial alleviation of epidermal hyperplasia, inflammatory cell infiltration, and stratum corneum exfoliation occurred at the highest dose (80 mg/kg) (Figure 7). The group receiving 60 mg/kg γ-linolenic acid (GLA) exhibited ear tissue morphology comparable to that of the group treated with 20 mg/kg of the BRB-F:BFR-F mixture.

Collectively, the histopathological improvements observed in treated mice corroborate the biochemical and immunological findings, demonstrating that oral administration of the BRB-F:BFR-F formulation leads to coordinated attenuation of epidermal hyperplasia, inflammatory infiltration, and structural disruption of the skin barrier. These tissue-level changes are consistent with modulation of early allergic signaling, Th1/Th2 rebalancing, and regulatory pathway engagement, reinforcing the concept of multi-axis immune regulation in the AD model.

## 3. Discussion

The present study demonstrates that bioprocessing substantially enhances the anti-atopic activities of both black rice bran and balloon flower root. Although the raw materials exhibited inherent but limited suppression of IgE synthesis, fermentation markedly amplified their bioactivity, consistent with previous reports that bioconversion improves the physiological functions and bioavailability of BRB-derived polysaccharides [16,17]. The similar degrees of inhibition produced by different BRB-F:BFR-F mixing ratios suggest that enhanced activity primarily reflects intrinsic properties of each extract rather than synergistic interactions, although the potential for synergy under alternative dosing conditions cannot be excluded. These observations further support the concept that fermentation-driven structural modification enhances the accessibility and functional potency of plant-derived immunoregulatory components.

Both raw and bioprocessed extracts were also capable of suppressing mast cell degranulation. The greater inhibition achieved with BRB and BRB-F compared with BFR and BFR-F indicates that mast cell-modulating constituents are more abundant or potent in black rice bran, which is consistent with previous observations that BRB-derived bioactives modulate mast cell-associated inflammatory signaling [15,17]. These results suggest that anti-degranulation activity relies largely on functional components already present in the raw materials rather than bioactive factors newly generated by fermentation.

In keratinocytes, bioprocessing enhanced suppression of pro-inflammatory cytokines and chemokines, mirroring the trends observed in IgE synthesis and mast cell activation. The stronger anti-inflammatory effect of BFR-F relative to BRB-F indicates that balloon flower root contains constituents that more strongly target keratinocyte-mediated inflammation, consistent with the known pharmacological activities of platycodin-containing extracts [32]. The lack of synergistic or additive effects across BRB-F:BFR-F mixing ratios suggests that the two materials act through complementary but largely independent biochemical pathways in this context.

In vivo, bioprocessing significantly enhanced the ability of both materials to attenuate AD pathology. Notably, efficacy increased progressively with the proportion of BRB-F in the formulation, supporting the interpretation that BRB-derived constituents make dominant contributions to moderating AD-associated immune dysregulation. This interpretation aligns with earlier reports demonstrating protective effects of BRB-derived bioactive compounds against allergic and inflammatory disorders [15,17]. The concomitant reductions in IgE, TSLP, IL-33 and IL-31 suggest action across multiple inflammatory axes rather than suppression of a single inflammatory pathway [33].

The BRB-F:BFR-F formulation modulated adaptive immunity in a bidirectional manner by attenuating Th2-dominant inflammation in lesional skin while restoring systemic Th1 cytokine expression. A similar pattern—where normalization of Th1 responses accompanies suppression of Th2 hyperactivation—has been reported in experimental models of AD treated with immunomodulatory natural products, supporting the concept of coordinated immune rebalancing rather than isolated pathway inhibition. These results indicate that the formulation restores immune balance rather than solely inhibiting inflammatory drivers [34].

Importantly, although IL-10 is generally considered an anti-inflammatory cytokine, elevated IL-10 levels have also been reported in patients with atopic dermatitis and other chronic allergic inflammatory conditions [4]. Such increases are thought to reflect a compensatory regulatory response to persistent immune activation rather than effective resolution of inflammation.

Bioprocessing also enhanced the ability of both extracts to inhibit IL-10 overexpression in vivo. Since dysregulated IL-10 secretion in Th2-dominant inflammatory environments has been associated with impaired Th1-mediated responses in AD [28], modulation of IL-10 expression may contribute to restoration of immune balance in this model. Likewise, induction of circulating galectin-9—an immunoregulatory factor associated with Treg activation and attenuation of Th2-dominant inflammation [29]—may represent an additional component of the therapeutic mechanism. Together, these findings suggest that BRB-F and BFR-F act through coordinated regulation of B-cell-, mast cell-, keratinocyte-, Th2-, Th1- and Treg-associated pathways, contributing both to immune rebalancing and improved tissue-level pathology.

Importantly, substantial therapeutic activity was observed even at relatively low doses. The 20 mg/kg treatment produced comparable or superior inhibitory activity relative to 60 mg/kg γ-linolenic acid, in agreement with previous findings showing that functional food-derived bioactive ingredients can serve as effective adjuncts in the management of AD [15,35,36,37]. Although the highest dose (80 mg/kg) yielded the strongest effects, the robust response at intermediate doses suggests that clinically meaningful benefits may be achievable without maximal exposure.

Collectively, the present findings support a model in which the BRB-F:BFR-F formulation exerts distributed regulation across multiple immune compartments, including early allergic effector mechanisms, epithelial inflammatory signaling, adaptive Th1/Th2 polarization, and regulatory immune pathways. Rather than functioning as a single-target inhibitor, the formulation appears to promote coordinated immune reorganization, consistent with the complex, multi-factorial pathogenesis of AD [38]. This systems-level regulatory profile may be particularly relevant in chronic inflammatory diseases characterized by immune heterogeneity and compartmental cross-talk.

This study has several limitations. First, all experiments were conducted using murine and in vitro experimental models, and therefore the observed effects are confined to preclinical systems. Second, although the present work demonstrates enhanced biological activity following bioprocessing, the specific bioactive molecules and fermentation-driven chemical transformations responsible for the observed immunomodulatory effects remain incompletely characterized. The chemical characteristics of BRB-F used in this study have been partially described in our previous work [17], in which γ-oryzanol was identified and quantified by HPLC analysis (4.11 mg/g) and the carbohydrate composition of the polysaccharide fraction was also examined. These previously reported compositional analyses support the reproducibility and chemical basis of the bioprocessed material used in the present study. However, comparable detailed characterization of BFR-F and direct compositional comparison of both bioprocessed materials within the present formulation have not yet been completed. In addition, because the extracts were administered through dietary supplementation under ad libitum feeding conditions, individual variation in food intake may have influenced the exact amount of extract consumed by each animal. Ongoing studies are focused on identifying the major immunologically active constituents of BFR-F and elucidating how bioprocessing alters the chemical profiles of both BRB-F and BFR-F. Such efforts will be essential for mechanistic understanding and for optimizing the formulation of this binary functional material. Because multiple intracellular signaling pathways may contribute to the observed immunomodulatory effects, future studies will investigate key signaling cascades, including NF-κB, MAPK, and JAK–STAT pathways, to further clarify the molecular mechanisms underlying the activity of this formulation.

## 4. Materials and Methods

### 4.1. Materials

An ointment containing DFE (Biostir, Hyogo, Japan) and DNCB (Sigma Chemical Co., St. Louis, MO, USA) was used as the antigen and hapten, respectively, for inducing AD-like skin lesions. Unless otherwise specified, all other reagents were obtained from Sigma (St. Louis, MO, USA). DNCB was dissolved in a solution of olive oil and acetone at a ratio of 3:1. The ELISA kit was purchased from R&D Systems (Minneapolis, MN, USA).

### 4.2. Preparation of Bioprocessed Black Rice Bran and Bioprocessed Balloon Flower Root

Black rice bran was obtained from JARM Agricultural Corporation (Jindo, Republic of Korea), and balloon flower root (*Platycodon grandiflorum*) was obtained from Chungbuk Yakcho Agricultural Cooperative (Jecheon Herb, Jecheon, Republic of Korea). The materials were commercially cultivated products and were not collected from the wild. Plant materials were obtained from certified commercial suppliers and identified based on supplier documentation and standard botanical references.

Bioprocessed (fermented) black rice bran (BRB-F) and bioprocessed (fermented) balloon flower root (BFR-F) were prepared according to previously reported methods [18]. *Lentinus edodes* mycelia were selected as the fermentation microorganism because fungal fermentation has been reported to enhance the release of bioactive compounds from plant matrices and improve the biological activity of plant-derived materials. Amylase was included to facilitate degradation of complex carbohydrate components and improve the accessibility of bioactive constituents during the bioprocessing process. *Lentinus edodes* mycelia grown on potato dextrose agar (PDA) were inoculated into 50 mL of liquid medium and incubated in 250 mL Erlenmeyer flasks at 28 °C for 5 days on a rotary shaker (120 rpm). The resulting pre-cultured mycelia were then used to inoculate the main liquid culture, which contained either black rice bran (100 g/L) or balloon flower root (100 g/L).

For enzymatic pretreatment, the culture was treated with amylase at 60 °C for 60 min. Fermentation proceeded in a 5 L fermenter (working volume 3 L) at 28 °C and 150 rpm with a 10% inoculum. After 3 days, the culture mass was subjected to additional enzymatic treatment using amylase and a mixed enzyme preparation to degrade particulate carbohydrate-rich material.

The culture was adjusted to pH 6.0 with HCl and subsequently sterilized in an autoclave. The main culture process was initiated by adding a cell-wall-degrading enzyme mixture containing cellulase, hemicellulase, pectinase, glucanase, mannose, and arabinase at 50 °C for 60 min. Following enzymatic treatment, the cultures were extracted with hot water at 90 °C for 1 h and then lyophilized to obtain the final solid bioprocessed materials.

### 4.3. U266.B1 Cell Culture and Measurement of IgE Production

These in vitro assays were selected to evaluate complementary mechanisms involved in allergic inflammation, including IgE production by B cells, mast cell degranulation, epithelial cytokine signaling, and keratinocyte-derived inflammatory mediators. In all in vitro experiments, untreated cells served as negative controls, while appropriate stimulation controls were used depending on the specific assay conditions. Measurement of IgE production using a B cell line was performed according to previously described methods [17]. The U266.B1 human multiple myeloma B lymphocyte cell line was obtained from the American Type Culture Collection (ATCC, Manassas, VA, USA). These cells were cultured in a modified RPMI1640 medium, supplemented with 10 mM HEPES, 2 mM L-glutamine, 1 mM sodium pyruvate, 4.5 g/L glucose, 1.5 g/L sodium bicarbonate, and 15% heat-inactivated fetal bovine serum (FBS). Penicillin (100 U/mL) and streptomycin (100 mg/mL) were also included in the medium to prevent bacterial contamination. The cells were maintained at 37 °C in a humidified atmosphere containing 5% CO_2_. The culture medium was replaced every three days, ensuring optimal conditions until the cells reached maximal density. To evaluate changes in IgE production, U266.B1 cells were seeded in 96-well plates at a density of 1 × 10^6^ cells per well in a total volume of 200 μL and incubated for 24 h. The cells were then stimulated with 10 μg/mL lipopolysaccharide (LPS), 5 ng/mL human IL-4, and the respective samples for an additional 72 h. After incubation, the supernatant was collected and transferred to centrifuge tubes. The samples were centrifuged at 12,000 rpm for 10 min to separate the culture supernatants. IgE levels in the supernatants were quantified using an ELISA kit (Cat. No. BMS2097, Invitrogen, Carlsbad, CA, USA) following the manufacturer’s protocol. The detection range of the assay was 7.8–500 ng/mL according to the manufacturer’s specifications. The absorbance of the reaction mixture was measured at 450 nm using a microplate reader (VersaMax, Molecular Devices Corp., San Jose, CA, USA).

### 4.4. RBL-2H3 Cell Culture and Measurement of β-Hexosaminidase Release

Measurement of β-hexosaminidase release using a mast cell line was performed according to previously described methods [17]. The RBL-2H3 rat basophilic leukemia mast cell line, obtained from ATCC, was cultured in a modified Dulbecco’s Modified Eagle Medium (DMEM). The medium was supplemented with 10 mM HEPES, 2 mM L-glutamine, 1 mM sodium pyruvate, 4.5 g/L glucose, 1.5 g/L sodium bicarbonate, and 10% heat-inactivated fetal bovine serum (FBS). To prevent bacterial contamination, penicillin (100 U/mL) and streptomycin (100 mg/mL) were added. The cells were maintained at 37 °C in a humidified incubator containing 5% CO_2_. The culture medium was replaced every 2 to 3 days, allowing the cells to proliferate until maximal density was achieved. To assess β-hexosaminidase activity, RBL-2H3 cells were seeded in 96-well plates at a density of 1 × 10^5^ cells per well in a total volume of 200 μL and incubated for 24 h. Each well received 200 μL of Tyrode buffer (137 mM NaCl, 2.7 mM KCl, 1.8 mM CaCl_2_, 1.1 mM MgCl_2_, 11.9 mM NaHCO_3_, 0.4 mM NaH_2_PO_4_, and 5.6 mM glucose, pH 7.2), along with the test samples, and was incubated for 15 min. After incubation, the extracts were removed by washing with Tyrode buffer. To stimulate the cells, calcium ionophore A23187 (10 μM) dissolved in Tyrode buffer was added for 20 min. Following stimulation, 50 μL of the supernatant containing released β-hexosaminidase was collected from each well. The collected supernatant was mixed with an equal volume of p-nitrophenyl-N-acetyl-β-glucosaminide solution (1 mM, pH 5.2) and incubated for 1 h at room temperature. The reaction was stopped by adding sodium carbonate buffer (67 mM, pH 10.2). The absorbance of the final mixture was measured at 405 nm using a microplate reader (VersaMax, Molecular Devices Corp., CA, USA), providing quantitative data on β-hexosaminidase release.

### 4.5. HMC-1.2 Cell Culture and Measurement of TSLP Production

Measurement of TSLP production was performed according to the method of Moon and Kim [39] with slight modifications. The HMC-1.2 human mast cell line, obtained from ATCC, was cultured using a modified Iscove’s Modified Dulbecco’s Medium (IMDM). The culture medium was supplemented with 10 mM HEPES, 2 mM L-glutamine, 1 mM sodium pyruvate, 4.5 g/L glucose, 1.5 g/L sodium bicarbonate, and 10% heat-inactivated fetal bovine serum (FBS). To prevent bacterial contamination, penicillin (100 U/mL) and streptomycin (100 mg/mL) were also included in the medium. The cells were maintained at 37 °C in a humidified atmosphere containing 5% CO_2_. The culture medium was replaced every 2 to 3 days to support cell proliferation until maximal density was reached. To assess changes in thymic stromal lymphopoietin (TSLP) production, HMC-1.2 cells were plated in 96-well plates at a density of 5 × 10^5^ cells per well in a total volume of 200 μL and incubated for 24 h. The samples to be tested were then added to each well, followed by a 2 h incubation period. Afterward, HMC-1.2 cells were stimulated with 50 μM phorbol 12-myristate 13-acetate (PMA) and 1 μg/mL calcium ionophore A23187 for 7 h to induce TSLP production. Following stimulation, the supernatant from each well was collected and transferred into centrifuge tubes. The samples were centrifuged at 12,000 rpm for 10 min to separate the culture supernatants. The TSLP levels in the supernatants were then measured using a specific ELISA kit (Cat. No. EHTSLP, Invitrogen) according to the manufacturer’s instructions. The detection range of the assay was 3.28–800 pg/mL according to the manufacturer’s specifications. The absorbance of the final reaction mixture was read at 450 nm using a microplate reader (VersaMax, Molecular Devices Corp., CA, USA), providing quantitative data on TSLP production.

### 4.6. HaCaT Cell Culture and Measurement of TARC, MDC, and IL-6 Production

Measurement of TARC, MDC, and IL-6 production using a keratinocyte cell line was performed according to the method of Park et al. [40] with slight modification. The HaCaT human keratinocyte cell line, obtained from the American Type Culture Collection (ATCC), was cultured using a modified Dulbecco’s Modified Eagle Medium (DMEM). The growth medium was supplemented with 10 mM HEPES, 2 mM L-glutamine, 1 mM sodium pyruvate, 4.5 g/L glucose, 1.5 g/L sodium bicarbonate, and 10% heat-inactivated fetal bovine serum (FBS). To prevent bacterial contamination, penicillin (100 U/mL) and streptomycin (100 mg/mL) were also added to the medium. The HaCaT cells were maintained at 37 °C in a humidified incubator containing 5% CO_2_. The culture medium was replaced every two to three days, supporting cell proliferation until maximal density was reached. For the assessment of chemokine and cytokine production, HaCaT cells were seeded into 48-well plates at a density of 4 × 10^5^ cells per well in a total volume of 400 μL and incubated for 24 h. After the initial incubation, the cells were further cultured for 24 h in serum-free DMEM. Test samples were then added to each well, and the cells were incubated for an additional 24 h. Following this treatment, HaCaT cells were stimulated with 10 ng/mL of TNF-α and IFN-γ for 24 h to induce the production of the target proteins. After stimulation, the culture supernatants were collected and transferred to centrifuge tubes. The samples were centrifuged at 12,000 rpm for 10 min to separate the supernatants. The concentrations of thymus and activation-regulated chemokine (TARC), macrophage-derived chemokine (MDC), and interleukin-6 (IL-6) in the supernatants were measured using specific ELISA kits (Cat. No. SDN00, DMD00, D6050; R&D Systems) according to the manufacturer’s protocols. The detection ranges of the assays were 31.2–2000 pg/mL for TARC, 125–4000 pg/mL for MDC, and 3.12–300 pg/mL for IL-6 according to the manufacturers’ specifications. The absorbance of the final reaction mixtures was determined at 450 nm using a microplate reader (VersaMax, Molecular Devices Corp., CA, USA), providing quantitative data for TARC, MDC, and IL-6 production.

### 4.7. Mice

Pathogen-free female BALB/c mice (6 weeks old, 18–20 g) were obtained from Koatech (Pyeongtaek, Republic of Korea). Upon arrival, the mice were housed in stainless-steel cages and maintained under a controlled environment featuring a 12 h light/dark cycle. The room temperature was kept at 23 °C with a margin of ±3 °C, and the relative humidity was regulated to 50 ± 10%. For the purpose of acclimation, the mice were provided with unrestricted access to pelletized standard commercial chow diet (Cat. No. 5L79, Orient Bio Inc., Seongnam, Republic of Korea) and tap water for one week. No injectable anesthetic agents were used in this study. All animals were euthanized by carbon dioxide (CO_2_) inhalation in accordance with institutional animal care and use guidelines. All animal procedures were approved by the Institutional Animal Care and Use Committee of the Chuncheon Bioindustry Foundation (CBF IACUC no. 2025-054; approval date: 16 September 2025) and were conducted in accordance with relevant guidelines and regulations.

### 4.8. DFE- and DNCB-Induced Allergic Dermatitis (AD) in Ear Skin

**Figure 8:**
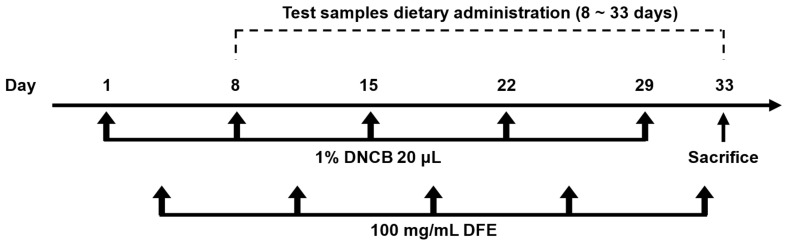
Induction of atopic dermatitis. Schematic diagram of the atopic dermatitis induction protocol. A total of 20 μL of 1% DNCB in olive oil/acetone (3:1) was applied to the front/back of the right ear (10 μL to each side) on Day 1, and 3 days later 100 mg/mL of DFE was applied to the same ear, with these treatments repeated weekly for five total treatments. One week after the initial challenge, test samples were administered orally for four weeks. Mice were sacrificed on Day 33, 24 h after the final treatment.

Allergic dermatitis-like lesions were induced in the ear skin of female BALB/c mice, following a modified protocol based on Choi et al. [15] and Hwang et al. [24]. The extract doses used in the present study were selected based on preliminary experiments and previously reported studies evaluating the biological activity of bioprocessed black rice bran and related plant-derived materials in murine models. The selected dose range allowed evaluation of both efficacy and dose-dependent responses without causing adverse effects in animals. Female mice were used to maintain consistency with previously reported murine models of atopic dermatitis and to reduce variability associated with sex-dependent immunological responses. The experimental procedure is illustrated in Figure 8. Two in vivo experiments were conducted in this study: (1) evaluation of the effects of individual extracts and their mixtures at different ratios, and (2) evaluation of the dose-dependent effects of the selected BRB-F:BFR-F formulation. The mice were randomly assigned to nine distinct groups (*n* = 10 per group): vehicle control; positive control (DFE/DNCB plus vehicle); DFE/DNCB plus black rice bran (BRB, 40 mg/kg); DFE/DNCB plus balloon flower root (BFR, 40 mg/kg); DFE/DNCB plus bioprocessed black rice bran (BRB-F, 40 mg/kg); DFE/DNCB plus bioprocessed balloon flower root (BFR-F, 40 mg/kg); and DFE/DNCB plus bioprocessed product mixtures in ratios of 3 BRB-F:1 BFR-F, 1 BRB-F:1 BFR-F, and 1 BRB-F:3 BFR-F (all at 40 mg/kg). To initiate the dermatitis model, each mouse received a topical application of 20 μL of 1% DNCB dissolved in an olive oil/acetone solution (3:1 ratio) to both the front and back of right ear. This was followed three days later by an application of DFE ointment (100 mg/mL). The combined DFE/DNCB exposure was repeated once weekly for five consecutive weeks. Successful induction of atopic dermatitis was confirmed by the appearance of characteristic clinical signs, including erythema and ear swelling, together with elevated serum IgE levels and histopathological changes such as epidermal thickening and inflammatory cell infiltration. One week after the initial challenge with DNCB and DFE, the mice began receiving the respective test samples administered orally by mixing the extracts with the diet for a total duration of four weeks, rather than by forced oral gavage. Daily food intake of the mice was monitored throughout the experimental period, and the average consumption was approximately 3 g per day. Based on this measured intake and the body weight of the animals, the experimental diets were formulated to provide the intended dose levels (mg/kg body weight). Food and water were supplied ad libitum during the study. At the end of the study period, all mice were sacrificed by CO_2_ inhalation, 24 h after the final treatment (day 33), to evaluate the atopic dermatitis-suppressive effects of bioprocessed black rice bran and bioprocessed balloon flower root. Following blood collection, the ears and spleen of each mouse were excised for subsequent histological analysis and ELISA assessment.

### 4.9. Measurement of Ear Thickness and Cytokines from Ear Tissue

After the mice were sacrificed, ear thickness was determined using a dial thickness gauge (Mitutoyo Corporation, Kanagawa, Japan) to quantitatively assess swelling. Ear tissues were homogenized in a phosphate buffer (pH 7) containing 0.4 M NaCl, 0.05% Tween-20, 0.5% bovine serum albumin (BSA), 0.1 mM phenylmethylsulfonyl fluoride (PMSF), and 10 mM ethylenediaminetetraacetic acid (EDTA). The resulting homogenates were then subjected to microcentrifugation at 14,000× *g* for 15 min at 4 °C to recover the supernatant, which contains the soluble cytokines. The concentrations of TSLP, IL-33, IL-31, and IL-10 in the ear tissue supernatants were subsequently measured using ELISA kits (R&D Systems, Minneapolis, MN, USA, and Thermo Fisher Scientific, Waltham, MA, USA) according to the manufacturers’ instructions. The detection ranges of the assays were 7.8–500 pg/mL for TSLP, 15.6–1000 pg/mL for IL-33, 15.6–1000 pg/mL for IL-31, and 15.6–1000 pg/mL for IL-10 according to the manufacturers’ specifications.

### 4.10. ELISA Measurement of Cytokine Levels in Serum

Blood samples were obtained from the sacrificed mice via cardiac puncture. Following collection, the samples were placed upright and allowed to rest for 30 min at 4 °C to facilitate clot formation of the red blood cells. Subsequently, the blood samples were centrifuged at 2000× *g* for 30 min at 4 °C using a Micro 17R centrifuge (Hanil Science, Incheon, Republic of Korea). The resulting supernatants were carefully collected and stored at −70 °C until further analysis. To quantify the levels of serum IgE and galectin-9, ELISA kits (R&D Systems, Minneapolis, MN, USA) were used according to the manufacturer’s instructions. The detection ranges of the assays were 6.25–400 ng/mL for IgE and 7.813–500 pg/mL for galectin-9 according to the manufacturers’ specifications.

### 4.11. RNA Isolation

Total RNA was extracted from biopsy specimens or mononuclear cells utilizing the protocol established by Chomczynski and Sacchi [41], with minor modifications tailored for this study. To achieve efficient cell lysis from tissue samples, forty consecutive cryostat sections, each 5 μM thick, were immersed in a buffer containing 4 M guanidinium isothiocyanate. Following the RNA extraction process, the isolated samples underwent treatment with DNase 1 (Promega Corp., Madison, WI, USA) for 30 min at 37 °C to eliminate any residual DNA. RNA purity and concentration were assessed by measuring the absorbance at 260 and 280 nm using a spectrophotometer, and samples with an A260/A280 ratio between 1.8 and 2.0 were considered acceptable for subsequent analysis. Throughout all enzymatic procedures involving RNA, an RNase inhibitor (Boehringer Mannheim Corp., Indianapolis, IN, USA) was consistently included to protect the integrity of the RNA.

### 4.12. Real-Time Polymerase Chain Reaction (qRT-PCR)

Quantitative real-time PCR (qRT-PCR) was performed using a Thermal Cycler Dice TP850 (Takarabio Inc., Shiga, Japan) in accordance with the manufacturer’s protocol. The reaction setup involved combining 2 μL of cDNA (containing 100 ng), 1 μL of a primer solution (sense and antisense, 0.4 μM each), 12.5 μL of SYBR Premix Ex Taq (Takarabio Inc.), and 9.5 μL of distilled water (dH_2_O), resulting in a total reaction volume of 25 μL per tube. The amplification protocol consisted of an initial denaturation step of 10 s at 95 °C, followed by 40 cycles comprising 5 s at 95 °C and 30 s at 60 °C. This was succeeded by additional steps: 15 s at 95 °C, 30 s at 60 °C, and a final 15 s at 95 °C. Quantification and normalization of mRNA expression levels were conducted using the TP850 software (version 5.11) provided by the manufacturer. Gene-specific primer sets used for qRT-PCR were commercially predesigned and purchased from OriGene Technologies Inc. (Rockville, MD, USA), and the primer information is summarized in Table 7. This ensured accurate assessment of gene expression in the analyzed samples.

### 4.13. Statistical Analysis

Results are expressed as the mean ± SD of three independent experiments. Significant differences between means were determined by one-way ANOVA followed by Duncan’s multiple range test using the Statistical Analysis System (SAS version 9.4, SAS Institute Inc., Cary, NC, USA). Prior to parametric statistical analysis, the data were examined to confirm approximate normal distribution. *p* < 0.05 was regarded as significant. Percent reductions were calculated using values obtained by subtracting the mean value obtained with vehicle from the mean values obtained using the positive control treatment and the experimental treatment.

## 5. Conclusions

The present study demonstrates that oral administration of a bioprocessed black rice bran (BRB-F) and balloon flower root (BFR-F) formulation attenuates atopic dermatitis-associated pathology in a murine model through coordinated modulation of multiple immune pathways. Dose-dependent suppression of epithelial alarmins, Th2-associated cytokines, and IgE production, together with partial restoration of Th1 responses and reinforcement of galectin-9-associated regulatory signaling, supports a model of multi-axis immune rebalancing rather than single-pathway inhibition.

These findings highlight the potential of fermentation-enhanced plant-derived materials to exert distributed immunomodulatory effects across early allergic signaling, adaptive immune polarization, and tissue-level inflammatory responses. Although the precise bioactive constituents responsible for these effects remain to be fully characterized—particularly in the case of bioprocessed balloon flower root—the present results provide mechanistic preclinical evidence supporting further investigation of this binary formulation as a multi-target immunoregulatory strategy for AD.

## Figures and Tables

**Table 1:** Inhibitory effect of IgE production in U266.B1 cells by combined ratios of BRB-F and BFR-F.

Treatment	IgE Concentration (ng/mL)
Vehicle	8.9 ± 0.6 ^e^
Positive control (10 μg/mL LPS + 5 ng/mL IL-4)	482.7 ± 31.7 ^a^
100 μg/mL BRB	396.8 ± 30.6 ^bc^
100 μg/mL BFR	431.9 ± 25.4 ^b^
100 μg/mL BRB-F	202.7 ± 15.2 ^d^
100 μg/mL BFR-F	205.4 ± 17.9 ^d^
75 μg/mL BRB-F + 25 μg/mL BFR-F (3:1)	204.3 ± 18.1 ^d^
50 μg/mL BRB-F + 50 μg/mL BFR-F (1:1)	198.3 ± 10.3 ^d^
25 μg/mL BRB-F + 75 μg/mL BFR-F (1:3)	209.6 ± 11.9 ^d^

Data are shown as the mean ± SD of three independent experiments. Values in each column with the same letter are not significantly different between groups at *p* < 0.05.

**Table 2:** Inhibitory effect of β-hexosaminidase releases in RBL-2H3 cells by combined ratios of BRB-F and BFR-F.

Treatment	β-Hexosaminidase Release (%)
Vehicle	0.0 ± 7.3 ^d^
Positive control (10 μM Calcium ionophore A23187)	100 ± 4.8 ^a^
100 μg/mL BRB	47.3 ± 2.6 ^c^
100 μg/mL BFR	68.5 ± 1.9 ^b^
100 μg/mL BRB-F	41.6 ± 4.5 ^c^
100 μg/mL BFR-F	66.1 ± 3.1 ^b^
75 μg/mL BRB-F + 25 μg/mL BFR-F (3:1)	44.8 ± 3.5 ^c^
50 μg/mL BRB-F + 50 μg/mL BFR-F (1:1)	44.1 ± 4.1 ^c^
25 μg/mL BRB-F + 75 μg/mL BFR-F (1:3)	46.4 ± 2.7 ^c^

Data are shown as the mean ± SD of three independent experiments. Values in each column with the same letter are not significantly different between groups at *p* < 0.05.

**Table 3:** Inhibitory effect of TSLP production in HMC-1 cells by combined ratios of BRB-F and BFR-F.

Treatment	TSLP Production (pg/mL)
Vehicle	36.3 ± 2.4 ^e^
Positive control (50 μM PMA + 1 μg/mL Calcium ionophore A23187)	82.9 ± 5.9 ^a^
100 μg/mL BRB	66.8 ± 4.3 ^c^
100 μg/mL BFR	74.1 ± 3.7 ^b^
100 μg/mL BRB-F	58.7 ± 3.6 ^d^
100 μg/mL BFR-F	68.3 ± 2.9 ^c^
75 μg/mL BRB-F + 25 μg/mL BFR-F (3:1)	59.6 ± 4.2 ^d^
50 μg/mL BRB-F + 50 μg/mL BFR-F (1:1)	62.9 ± 5.6 ^cd^
25 μg/mL BRB-F + 75 μg/mL BFR-F (1:3)	66.2 ± 5.1 ^c^

Data are shown as the mean ± SD of three independent experiments. Values in each column with the same letter are not significantly different between groups at *p* < 0.05.

**Table 4:** Inhibitory effect of TARC, MDC, and IL-6 production in HaCaT cells by combined ratios of BRB-F and BFR-F.

Treatment	Cytokine and Chemokine Production (pg/mL)		
	TARC	MDC	IL-6
Vehicle	12.0 ± 3.7 ^i^	33.5 ± 14.3 ^h^	6.6 ± 1.3 ^g^
Positive control (10 ng/mL TNF-α/IFN-γ)	341.7 ± 6.8 ^a^	2999.3 ± 253.3 ^a^	109.7 ± 2.7 ^a^
100 μg/mL BRB	218.0 ± 16.5 ^c^	1703.8 ± 134.7 ^c^	66.1 ± 3.9 ^c^
100 μg/mL BFR	268.1 ± 13.6 ^b^	2421.1 ± 150.7 ^b^	81.4 ± 2.6 ^b^
100 μg/mL BRB-F	130.2 ± 6.4 ^d^	942.2 ± 64.5 ^d^	52.5 ± 3.4 ^ef^
100 μg/mL BFR-F	30.1 ± 6.9 ^h^	600.1 ± 59.6 ^g^	61.4 ± 2.4 ^d^
75 μg/mL BRB-F + 25 μg/mL BFR-F (3:1)	97.4 ± 18.5 ^e^	892.4 ± 80.4 ^de^	50.2 ± 1.0 ^f^
50 μg/mL BRB-F + 50 μg/mL BFR-F (1:1)	55.5 ± 2.7 ^f^	791.7 ± 31.9 ^e^	51.9 ± 1.2 ^f^
25 μg/mL BRB-F + 75 μg/mL BFR-F (1:3)	44.8 ± 4.1 ^g^	717.3 ± 51.2 ^f^	55.0 ± 2.2 ^e^

Data are shown as the mean ± SD of three independent experiments. Values in each column with the same letter are not significantly different between groups at *p* < 0.05.

**Table 5:** Regulatory effect of Th1 and Th2 cytokines in atopic dermatitis-induced mice by combined ratios of BRB-F and BFR-F.

Treatment	Cytokine Production					
	Th2 Cytokine mRNA in Ear (Fold-Increase)			Th1 Cytokine mRNA in Spleen (Fold-Increase)		
	IL-4	IL-5	IL-13	IL-2	IL-12	IFN-γ
Vehicle	1 ± 0.07 ^f^	1 ± 0.06 ^f^	1 ± 0.03 ^g^	1 ± 0.06 ^a^	1 ± 0.07 ^a^	1 ± 0.09 ^a^
Positive control	5.41 ± 0.46 ^a^	5.93 ± 0.41 ^a^	5.69 ± 0.43 ^a^	0.75 ± 0.05 ^d^	0.62 ± 0.04 ^d^	0.71 ± 0.05 ^d^
40 mg/kg BRB	5.03 ± 0.37 ^b^	5.13 ± 0.25 ^b^	5.16 ± 0.43 ^b^	0.77 ± 0.07 ^cd^	0.65 ± 0.05 ^d^	0.71 ± 0.06 ^d^
40 mg/kg BFR	5.24 ± 0.3 ^ab^	5.25 ± 0.29 ^b^	5.38 ± 0.39 ^ab^	0.74 ± 0.03 ^d^	0.63 ± 0.02 ^d^	0.72 ± 0.04 ^d^
40 mg/kg BRB-F	3.98 ± 0.31 ^cd^	4.09 ± 0.29 ^d^	4.21 ± 0.33 ^cd^	0.85 ± 0.04 ^bc^	0.82 ± 0.06 ^bc^	0.77 ± 0.03 ^c^
40 mg/kg BFR-F	4.27 ± 0.28 ^c^	4.55 ± 0.28 ^c^	4.49 ± 0.36 ^c^	0.81 ± 0.05 ^c^	0.8 ± 0.07 ^bc^	0.76 ± 0.06 ^cd^
40 mg/kg BRB-F + BFR-F (3:1)	3.26 ± 0.25 ^e^	3.51 ± 0.22 ^e^	3.36 ± 0.16 ^f^	0.94 ± 0.07 ^ab^	0.88 ± 0.08 ^b^	0.85 ± 0.06 ^b^
40 mg/kg BRB-F + BFR-F (1:1)	3.55 ± 0.17 ^de^	3.72 ± 0.41 ^de^	3.7 ± 0.24 ^de^	0.89 ± 0.08 ^b^	0.86 ± 0.05 ^b^	0.82 ± 0.07 ^bc^
40 mg/kg BRB-F + BFR-F (1:3)	3.71 ± 0.29 ^d^	3.86 ± 0.35 ^de^	3.94 ± 0.28 ^d^	0.86 ± 0.05 ^bc^	0.83 ± 0.03 ^bc^	0.8 ± 0.05 ^bc^

Data are shown as the mean ± SD of three independent experiments. Values in each column with the same letter are not significantly different between groups at *p* < 0.05.

**Table 6:** Dose-dependent effects of BRB-F and BFR-F (3:1) on Th1 and Th2 cytokines.

Treatment	Cytokine Production					
	Th2 Cytokine mRNA in Ear (Fold-Increase)			Th1 Cytokine mRNA in Spleen (Fold-Increase)		
	IL-4	IL-5	IL-13	IL-2	IL-12	IFN-γ
Vehicle	1 ± 0.06 ^d^	1 ± 0.07 ^d^	1 ± 0.06 ^d^	1 ± 0.03 ^a^	1 ± 0.06 ^a^	1 ± 0.05 ^a^
Positive	5.59 ± 0.48 ^a^	6.03 ± 0.51 ^a^	5.79 ± 0.52 ^a^	0.62 ± 0.02 ^e^	0.71 ± 0.05 ^c^	0.75 ± 0.05 ^c^
10 mg/kg	5.13 ± 0.38 ^ab^	5.48 ± 0.53 ^ab^	5.29 ± 0.48 ^b^	0.65 ± 0.05 ^de^	0.73 ± 0.06 ^c^	0.77 ± 0.03 ^c^
20 mg/kg	4.83 ± 0.41 ^b^	5.12 ± 0.50 ^b^	5.05 ± 0.41 ^b^	0.72 ± 0.04 ^d^	0.76 ± 0.07 ^c^	0.80 ± 0.05 ^bc^
40 mg/kg	3.77 ± 0.31 ^c^	4.19 ± 0.40 ^c^	4.06 ± 0.32 ^c^	0.81 ± 0.05 ^bc^	0.83 ± 0.06 ^b^	0.86 ± 0.06 ^b^
80 mg/kg	3.51 ± 0.28 ^c^	4.06 ± 0.35 ^c^	3.82 ± 0.31 ^c^	0.85 ± 0.06 ^b^	0.87 ± 0.07 ^b^	0.89 ± 0.05 ^b^
GLA	4.93 ± 0.36 ^b^	5.34 ± 0.41 ^ab^	5.16 ± 0.49 ^b^	0.69 ± 0.06 ^d^	0.75 ± 0.08 ^c^	0.78 ± 0.07 ^c^

Data are shown as the mean ± SD of three independent experiments. Values in each column with the same letter are not significantly different between groups at *p* < 0.05.

**Table 7:** Commercially predesigned primer sets used for qRT-PCR analysis.

Gene	Supplier	Catalog No.
IL-4	OriGene Technologies	MP206793
IL-5	OriGene Technologies	MP206796
IL-13	OriGene Technologies	MP206748
IL-2	OriGene Technologies	MP206769
IL-12	OriGene Technologies	MP206746
IFN-γ	OriGene Technologies	MP206683
IL-10	OriGene Technologies	MP206737
GAPDH	OriGene Technologies	MP205604

## Data Availability

The original contributions presented in this study are included in the article. Further inquiries can be directed to the corresponding authors.

## References

[1] Guttman-Yassky E., Renert-Yuval Y., Brunner P.M. (2025). Atopic dermatitis. Lancet.

[2] Sroka-Tomaszewska J., Trzeciak M. (2021). Molecular mechanisms of atopic dermatitis pathogenesis. Int. J. Mol. Sci..

[3] Langan S.M., Irvine A.D., Weidinger S. (2020). Atopic dermatitis. Lancet.

[4] Brunner P.M., Guttman-Yassky E., Leung D.Y.M. (2017). The immunology of atopic dermatitis and its reversibility with broad-spectrum and targeted therapies. J. Allergy Clin. Immunol..

[5] Thyssen J.P., Halling A.S., Schmid-Grendelmeier P., Guttman-Yassky E., Silverberg J.I. (2023). Comorbidities of atopic dermatitis—What does the evidence say?. J. Allergy Clin. Immunol..

[6] Wollenberg A., Christen-Zäch S., Taieb A., Paul C., Thyssen J.P., de Bruin-Weller M., Vestergaard C., Seneschal J., Werfel T., Cork M.J. (2020). ETFAD/EADV eczema task force 2020 position paper on diagnosis and treatment of atopic dermatitis in adults and children. J. Eur. Acad. Dermatol. Venereol..

[7] Sidbury R., Davis D.M., Cohen D.E., Cordoro K.M., Berger T.G., Bergman J.N., Chamlin S.L., Cooper K.D., Feldman S.R., Hanifin J.M. (2014). Guidelines of care for the management of atopic dermatitis: Section 1. Diagnosis and assessment of atopic dermatitis. J. Am. Acad. Dermatol..

[8] Calder P.C. (2013). Feeding the immune system. Proc. Nutr. Soc..

[9] Tollefson M.M., Bruckner A.L. (2014). Section on Dermatology. Atopic dermatitis: Skin-directed management. Pediatrics.

[10] Powell N., Walker M.M., Talley N.J. (2017). The mucosal immune system: Master regulator of bidirectional gut–brain communications. Nat. Rev. Gastroenterol. Hepatol..

[11] Gittler J.K., Shemer A., Suárez-Fariñas M., Fuentes-Duculan J., Gulewicz K.J., Wang C.Q.F., Mitsui H., Cardinale I., de Guzman Strong C., Krueger J.G. (2012). Progressive activation of Th2/Th22 cytokines and selective epidermal proteins characterizes acute and chronic atopic dermatitis. J. Allergy Clin. Immunol..

[12] Kubo A., Nagao K., Amagai M. (2012). Epidermal barrier dysfunction and cutaneous sensitization in atopic dermatitis. J. Clin. Investig..

[13] Belkaid Y., Segre J.A. (2014). Dialogue between skin microbiota and immunity. Science.

[14] Huang Y.P., Lai H.M. (2016). Bioactive compounds and antioxidative activity of colored rice bran. J. Food Drug Anal..

[15] Choi S.P., Kim S.P., Kang M.Y., Nam S.H., Friedman M. (2010). Protective effects of black rice bran against chemically-induced inflammation of mouse skin. J. Agric. Food Chem..

[16] Kim S.P., Park S.O., Lee S.J., Nam S.H., Friedman M. (2013). A polysaccharide isolated from the liquid culture of Lentinus edodes mushroom mycelia containing black rice bran protects mice against a Salmonella lipopolysaccharide-induced endotoxemia. J. Agric. Food Chem..

[17] Kwon K.S., Hwang W.S., Lee K.H., Kim K.J., Lee W.Y., Kim J., Lee S.J., Kim S.P., Friedman M. (2023). Protection of allergic asthma in mice by black rice bran bioprocessed with shiitake mushroom mycelia. Food Nutr. Sci..

[18] Kwon K.S., Lee E.S., Lee K.H., Hwang W.S., Lee W.Y., Kim J.J., Kim J., Lee S.J., Kim S.P., Friedman M. (2024). Anti-obesity and other health benefits of bioprocessed black rice bran in combination with green tea extract in 3T3-L1 preadipocyte cells and in mice on a high-fat diet. Food Funct..

[19] Lee K.H., Kwon K.S., Hwang W.S., Lee W.Y., Kim J., Lee S.J., Kim S.P., Friedman M. (2023). Bioprocessed black rice bran potentiates the growth inhibitory activity of an immune checkpoint inhibitor against murine colon carcinoma. Food Nutr. Sci..

[20] Hur S.J., Lee S.Y., Kim Y.C., Choi I., Kim G.B. (2014). Effect of fermentation on the antioxidant activity in plant-based foods. Food Chem..

[21] Marco M.L., Heeney D., Binda S., Cifelli C.J., Cotter P.D., Foligné B., Gänzle M., Kort R., Pasin G., Pihlanto A. (2017). Health benefits of fermented foods: Microbiota and beyond. Curr. Opin. Biotechnol..

[22] Zhang L., Wang Y., Yang D., Zhang C., Zhang N., Li M., Liu Y. (2015). Platycodon grandiflorus—An ethnopharmacological, phytochemical and pharmacological review. J. Ethnopharmacol..

[23] Zhang L., Wang X., Zhang J., Liu D., Bai G. (2024). Ethnopharmacology, phytochemistry, pharmacology and product application of *Platycodon grandiflorum*: A review. Chin. Herb. Med..

[24] Xie L., Zhao Y.X., Zheng Y., Li X.F. (2023). The pharmacology and mechanisms of platycodin D, an active triterpenoid saponin from *Platycodon grandiflorus*. Front. Pharmacol..

[25] Tamang J.P., Watanabe K., Holzapfel W.H. (2016). Diversity of microorganisms in global fermented foods and beverages. Front. Microbiol..

[26] Jung J.I., Lee H.S., Kim S.M., Kim S., Lim J., Woo M., Kim E.J. (2022). Immunostimulatory activity of hydrolyzed and fermented *Platycodon grandiflorum* extract occurs via the MAPK and NF-κB signaling pathway in RAW 264.7 cells. Nutr. Res. Pract..

[27] Hwang J.S., Kwon H.K., Kim J.E., Rho J., Im S.H. (2012). Immunomodulatory effect of water-soluble extract separated from mycelium of Phellinus linteus on experimental atopic dermatitis. BMC Complement. Altern. Med..

[28] Oyoshi M.K., Larson R.P., Ziegler S.F., Geha R.S. (2010). Mechanical injury polarizes skin dendritic cells to elicit a TH2 response by inducing cutaneous thymic stromal lymphopoietin expression. J. Allergy Clin. Immunol..

[29] Su W., Zhang J., Yang S., Tang M., Shen Y., Liu C., Ji J., Maurer M., Jiao Q. (2022). Galectin-9 contributes to the pathogenesis of atopic dermatitis via T cell immunoglobulin mucin-3. Front. Immunol..

[30] Kim Y.J., Choi M.J., Bak D.H., Lee B.C., Ko E.J., Ahn G.R., Ahn S.W., Kim M.J., Na J., Kim B.J. (2018). Topical administration of EGF suppresses immune response and protects skin barrier in DNCB-induced atopic dermatitis in NC/Nga mice. Sci. Rep..

[31] Lee J.H., Lee Y.J., Lee J.Y., Park Y.M. (2017). Topical application of eupatilin ameliorates atopic dermatitis-like skin lesions in NC/Nga mice. Ann. Dermatol..

[32] Zhao J.Y., Guo J., Ye H.Y., Luo P.W., Zhu Q., Xu H., Zhou Y., Wang Y.J. (2026). Structure–function relationships of edible and medicinal mushroom polysaccharides: Structural analysis, target molecules and signaling pathways. Carbohydr. Polym..

[33] Wang Z.Y., Zheng Y.X., Xu F., Cui Y.Z., Chen X.Y., Chen S.Q., Yan B.X., Zhou Y., Zheng M., Man X.Y. (2023). Epidermal keratinocyte-specific STAT3 deficiency aggravates atopic dermatitis-like skin inflammation in mice through TLSP upregulation. Front. Immunol..

[34] Facheris P., Jeffery J., Del Duca E., Guttman-Yassky E. (2023). The translational revolution in atopic dermatitis: The paradigm shift from pathogenesis to treatment. Cell. Mol. Immunol..

[35] Livshits G., Kalinkovich A. (2024). Resolution of chronic inflammation, restoration of epigenetic disturbances and correction of dysbiosis as an adjunctive approach to the treatment of atopic dermatitis. Cells.

[36] Khan A., Adalsteinsson J., Whitaker-Worth D.L. (2022). Atopic dermatitis and nutrition. Clin. Dermatol..

[37] Rustad A.M., Nickles M.A., Bilimoria S.N., Lio P.A. (2022). The role of diet modification in atopic dermatitis: Navigating the complexity. Am. J. Clin. Dermatol..

[38] Criado P.R., Miot H.A., Bueno-Filho R., Ianhez M., Criado R.F.J., Castro C.C.S. (2024). Update on the pathogenesis of atopic dermatitis. An. Bras. Dermatol..

[39] Moon P.D., Kim H.M. (2011). Thymic stromal lymphopoietin is expressed and produced by caspase-1/NF-κB pathway in mast cells. Cytokine.

[40] Park J.W., Oh J., Hwang D., Kim S.M., Min J.H., Seo J.Y., Chun W., Lee H.J., Oh S.R., Lee J.W. (2021). 3,4,5-Trihydroxycinnamic acid exerts anti-inflammatory effects on TNF-α/IFN-γ-stimulated HaCaT cells. Mol. Med. Rep..

[41] Chomczynski P., Sacchi N. (1987). Single-step method of RNA isolation by acid guanidinium thiocyanate-phenol-chloroform extraction. Anal. Biochem..

